# Multifunctional self-assembled monolayers via microcontact printing and degas-driven flow guided patterning

**DOI:** 10.1038/s41598-018-35195-9

**Published:** 2018-11-13

**Authors:** Sang Hun Lee, Won-Yeop Rho, Seon Joo Park, Jinyeong Kim, Oh Seok Kwon, Bong-Hyun Jun

**Affiliations:** 1 School of Chemical & Biological Engineering, Seoul National University, Seoul, 00826 Republic of Korea; 2 School of International Engineering and Science, Chonbuk National University, Jeonju, 54896 Republic of Korea; 3 Harzards Monitoring Bionano Research Center, Korea Research Institute of Bioscience and Biotechnology (KRIBB), Daejeon, 34141 Republic of Korea; 4 Department of Bioscience and Biotechnology, Konkuk University, Seoul, 05029 Republic of Korea

## Abstract

Soft lithography-based patterning techniques have been developed to investigate biological and chemical phenomena. Until now, micropatterning with various materials required multiple procedural steps such as repeating layer-by-layer patterning, aligning of stamps, and incubating printed inks. Herein, we describe a facile micropatterning method for producing chemically well-defined surface architectures by combining microcontact (µCP) and microfluidic vacuum-assisted degas-driven flow guided patterning (DFGP) with a poly(dimethylsiloxane) (PDMS) stamp. To demonstrate our concept, we fabricated a bi-composite micropatterned surface with different functional molecular inks such as fluorescein isothiocyanate labelled bovine serum albumin (FITC-BSA) and polyethylene glycol (PEG)-silane for a biomolecule array, and 3-aminopropyltriethoxysilane (APTES) and PEG-silane pattern for a self-assembled colloid gold nanoparticle monolayer. With a certain composition of molecular inks for the patterning, bi-composite surface patterns could be produced by this µCP-DFGP approach without any supplementary process. This patterning approach can be used in microfabrication and highly applicable to biomolecules and nanoparticles that spread as a monolayer.

## Introduction

Micropatterns are now widely used in various research fields such as tissue engineering, developing bio-chips or sensors, etc^1–4^. The creation of a micropattern with a complex architecture typically requires multiple processing steps, specialized facilities, and harsh conditions, including metal deposition and dry/wet etching^5^. Conventional microfabrication techniques, such as soft lithography^6^, e-beam lithography^7,8^, dip-pen lithography^9^, nanoimprint lithography^10^, and ink jet printing^11–13^ have been widely adopted to produce well-defined micropatterns. Yet, a non-conventional microscale patterning method, such as microcontact printing (µCP), can still offer additional advantages of low cost, operation compatibility and flexibility^14–16^, and as a result, the µCP technique has become a routine method to generate micropatterns with a large variety of desired functional agents in chemical and biological research fields. Recent studies have focused on new patterning methods to provide well-defined surface architectures such as the biomolecule micropatterns, multicolour fluorescence patterns and the directed self-assembled monolayers (SAMs) to tailor nanotopography with high-resolution^17–19^. Iterative microcontact printing was demonstrated to fabricate a multilevel fluorescent tag, which consists of two overlapping micropatterns by the additive grafting of different functional molecule in the same area^20,21^. The spontaneous self-assembly of fiber-forming oligothiophenes via lithographically controlled wetting (LCW) technique was also described to construct the well-controlled semiconducting fiber for organic field-effect transistor (OFET) devices^22^. Ravoo group describes the micromolding in capillaries (MIMIC)-based patterned surfaces which have both protein adhesive and repellent areas^23,24^. Haman *et al*., also reported the printing and vacuum lithography (PnV lithography) for multiple AuNP patterns in the single plane to study the cellular morphology and adhesion^25^. However, although the appearance of numerous alternatives developed for biomolecules and self-assembled monolayers (SAM) pattern, rapid and facile micropattern preparation method still required for the various surface-related applications. However, this method requires several factors to consider-tuning of the wettability of stamp substrates prior to µCP, applying a constant pressure to stamp during µCP, controlling the duration of stamping and performing multiple procedures to generate complex patterns if different molecules are employed^26^. Furthermore, layer-by-layer deposition via µCP involves repeated sequential µCP and incubation of printing ink.

Over the past decade, microfluidic patterning methods via pressure- and electrokinetic-driven pumping have been developed^27–29^. Pressure-driven pumping can be accomplished with either a positive or a negative pressure, which pushes or pulls fluids into a microfluidic channel^30^. Alternatively, electrokinetic-driven pumping can utilize electric field generated by integrated electrodes in fluidic actuation^31^. The microfluidic patterning of both methods are commonly used to supply patterning inks or reagents, but they rely on external power sources and equipment such as a syringe pump. A capillary-guided patterning (CGP) method has been reported which offers simple and robust procedures for producing 3D hydrogels yet with some limitations, including multiple injection of different molecules and precise control of micropump for liquid aspiration^1^. Compared to other microfluidic patterning methods, degas-driven flow guided patterning (DFGP) offers several benefits: (i) simple operation and scalability in large area patterning; (ii) flexibility in terms of pattern shape, size, and materials; and (iii) no requirement for special equipment, complex process and external power source.

Herein, we report a facile micropatterning method via combined µCP and DFGP for cost- and process-effective fabrication of micropatterns. This technique is based on µCP with vacuum-assisted DFGP using unique property of PDMS. After completion of a µCP process with 1^st^ ink, 2^nd^ ink can be aspired by DFGP through microfluidic channels in the free space of a PDMS stamp. Also, a variety of micropatterns can be produced for diverse purposes without supplementary processes. As a proof of our concept, we have demonstrated that bi-composite architectures were patterned for selective immobilization of protein and gold nanoparticles (AuNPs)^3^. Our approach is simple, convenient and inexpensive, which can be used to print a variety of SAMs.

## Results and Discussion

**Figure 1:**
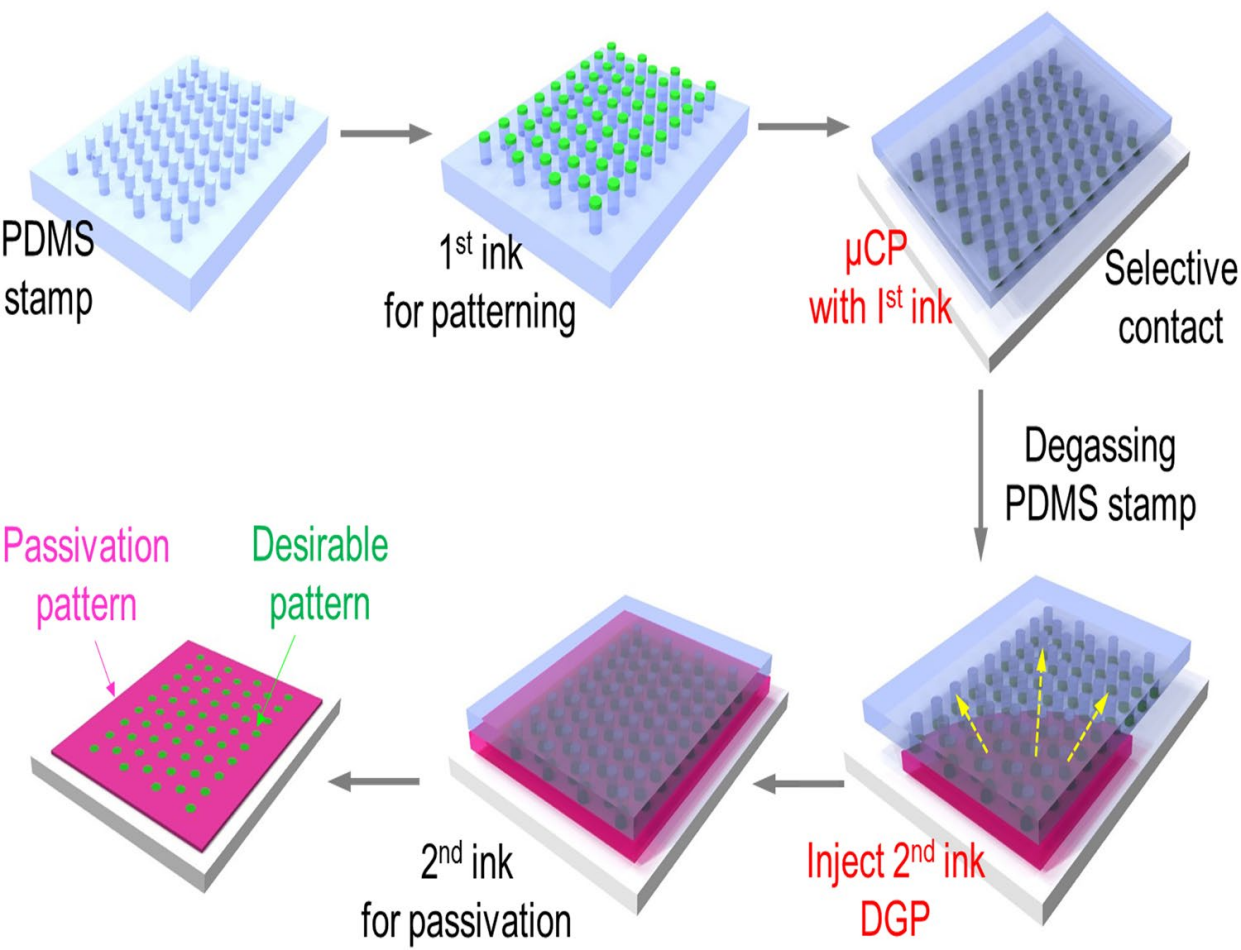
Schematic illustration of the process for fabricating a micro-patterned assay via simultaneous microcontact printing (μCP) and degas-driven flow-guided patterning (DFGP). Step 1. Soaking of 1^st^ molecular ink to a PDMS stamp for formation of desired 1^st^ patterns and then conformal contacting to glass slide for μCP; Step 2. Degassing the porous PDMS stamp to remove the air; Step 3. Patterning with 2^nd^ ink into free surface of the PDMS stamp through DFGP; and Step 4. Removal of the PDMS stamp and observation of a micropatterned array with functional molecules on the substrate.

Figure 1 shows a procedure to produce a micro-pattern by sequential µCP and DFGP. Photolithography was used to generate a master mould of a PDMS stamp for µCP. A micropattern in a silicon wafer was created using selective exposure of a thin film of a light-sensitive organic polymer (photoresist) to UV light, generating an inverse master mould. The aspect ratio (length or radius/height) of the relief microstructures was 1.4 (circular pattern: 70 µm in diameter and 50 µm in height) to withstand compressive forces during printing by mechanical distortions of the stamp^32^. Next, we produced a patterned surface via a sequential µCP-DFGP approach as shown in Fig. 1. Prior to 1^st^ ink patterning, the PDMS stamp was soaked in a fluorescein isothiocyanate (FITC)-labelled BSA (FITC-BSA) and then placed on a glass slide for 5 min. After that the PDMS stamp was degassed in a vacuum desiccator for 15 min, and the 2^nd^ ink for passivation was dispensed at the opening of an inlet with a micropipette. After 1 h incubation, the PDMS stamp was removed and rinsed 3 times. The patterned glass was then observed by a fluorescence microscopy. Two interactions were critical for µCP-DFGP. One was the interaction between the PDMS stamp and the 1^st^ ink, and the other was the adhesion between the ink and the substrate. Since unmodified PDMS is highly hydrophobic, the hydrophobic surface of PDMS was readily modified to a hydrophilic surface by oxygen plasma treatment to permit easy transfer of the 1^st^ ink from the PDMS to the substrate. In addition, we capitulated the use of DFGP based on unique characteristics of the PDMS which is a solubility of gas for fluid actuation without any surface modification of substrate for capillary force-driven flow.

**Figure 2:**
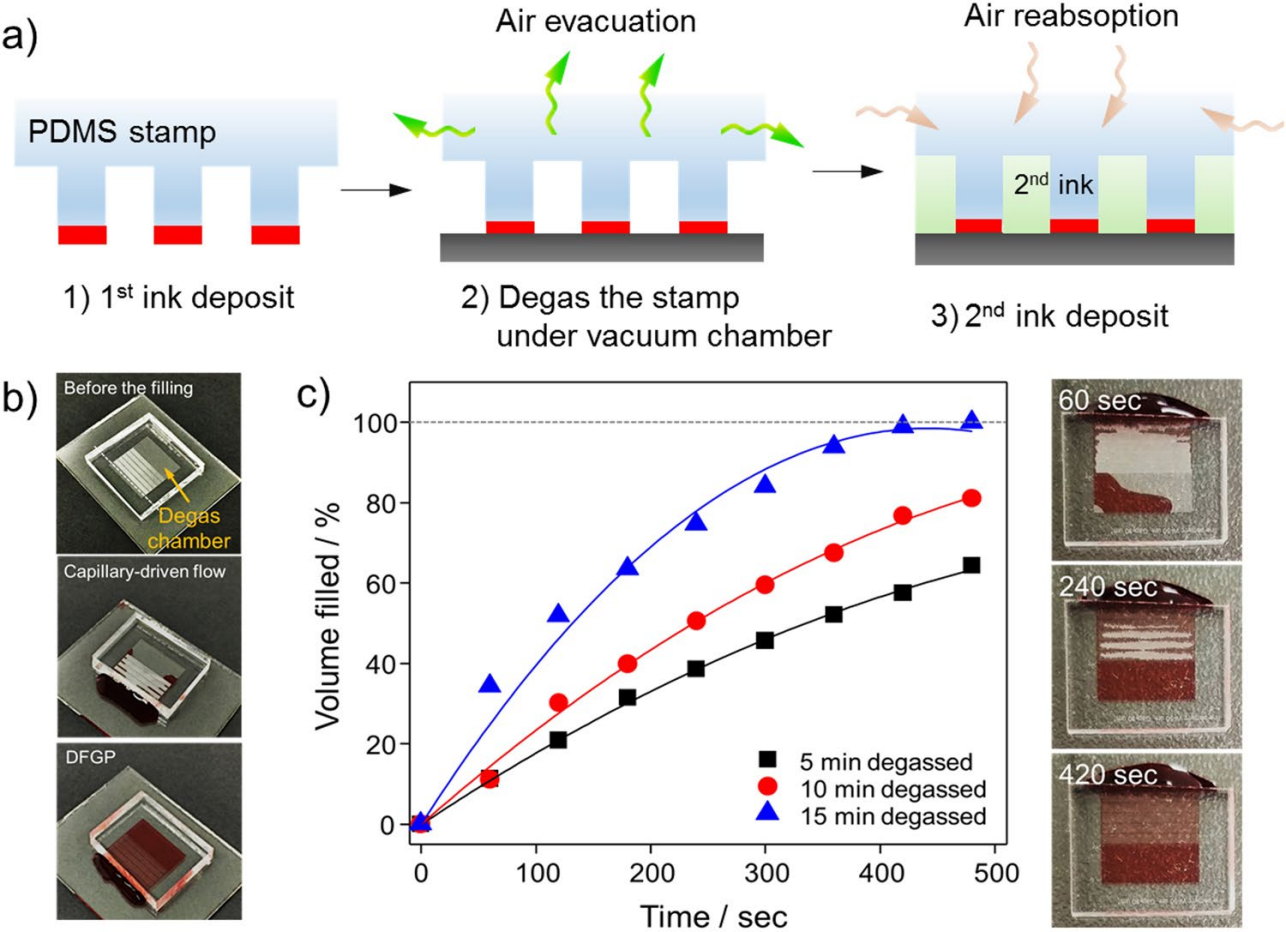
Working principle of degas-driven flow guided patterning (DFGP). (**a**) Schematic illustration of the DFGP procedure for self-transportation of μstamping ink. The proposed mechanism of the degassed PDMS stamp is based on its high air solubility. (i) PDMS stamp covered with 1^st^ ink; (ii) degassing of the PDMS stamp in a vacuum chamber; (iii) removing the PDMS stamp from vacuum conditions and placing 2^nd^ ink on an inlet; and (iv) aspiration of the 2^nd^ ink into the free cavity of the PDMS stamp under negative pressure created by the degassed PDMS. (**b**) Optical micrographs of microfluidic μstamp filled with red-color dye. (**c**) Different degassing time were tested (5 min to 15 min) to generate the degas flow-based fluidic actuation for DFGP.

Figure 2a depicts a schematic representation of sequential µCP and DFGP in producing desirable micropatterns. To overcome a capillary barrier of the surface, we adapted DFGP for strong fluidic actuation as alternative capillary-driven flow to produce topographic and biological/chemical patterns. It is known that PDMS is a porous elastomer, thus it has the property of reversible gas solubility of PDMS, obeying Henry’s law^33^.The degas-driven pumping technique is based on gas solubility of PDMS, and the following paragraph describes the working principle of DFGP. At atmospheric pressure, PDMS contains a large amount of air, which can be evacuated under a vacuum to a low gas solubility condition. The equilibrium concentration of gas in the PDMS is essentially proportional to the partial pressure of gas in the degassed PDMS^34^. When the degassed PDMS is brought back to atmospheric pressure, it will reabsorb air into the PDMS toward an original equilibrium state. The air re-absorption reduces the pressure inside microchannels when the inlets are plugged with aqueous solution droplets. Finally, the aqueous solution is pulled by negative pressure through the free cavity of the PDMS stamp.

Figure 2b (top image) shows optical micrographs of the PDMS stamp. The capillary action/surface tension-based fluid propulsion requires hydrophilic surfaces to generate flow of aqueous solutions. However, the PDMS stamp has numerous 3D microchannel structures which have hydrophobic property, thus it can play a key role in creating a hydrophobic barrier to suppress the capillary action (Fig. 2b, middle). Therefore, DFGP is more suitable to transport the ink to desirable areas than the capillary-driven flow as represented in Fig. 2b (bottom). To ensure sufficient fluidic actuation, DFGP method was utilized rather than the capillary driven flow. In order to generate the fluidic actuation for DFGP, we characterized the effect of an optimal t_d_ (the time the PDMS µStamp is stored in low pressure) on the device filling rate. The flow rate during the device priming can be controlled thought the degassing time. A td as low as 15 min can generate enough degas-driven flow to completely fill the device with the 2^nd^ ink within 10 min (Fig. 2d). The results shown in Fig. 2c, the flow rate can be increased by a prolong degassing time. In addition, our DFGP takes an advantage of the elimination of air bubble traps during the priming of pattering ink because of the use of gas solubility in PDMS.

**Figure 3:**
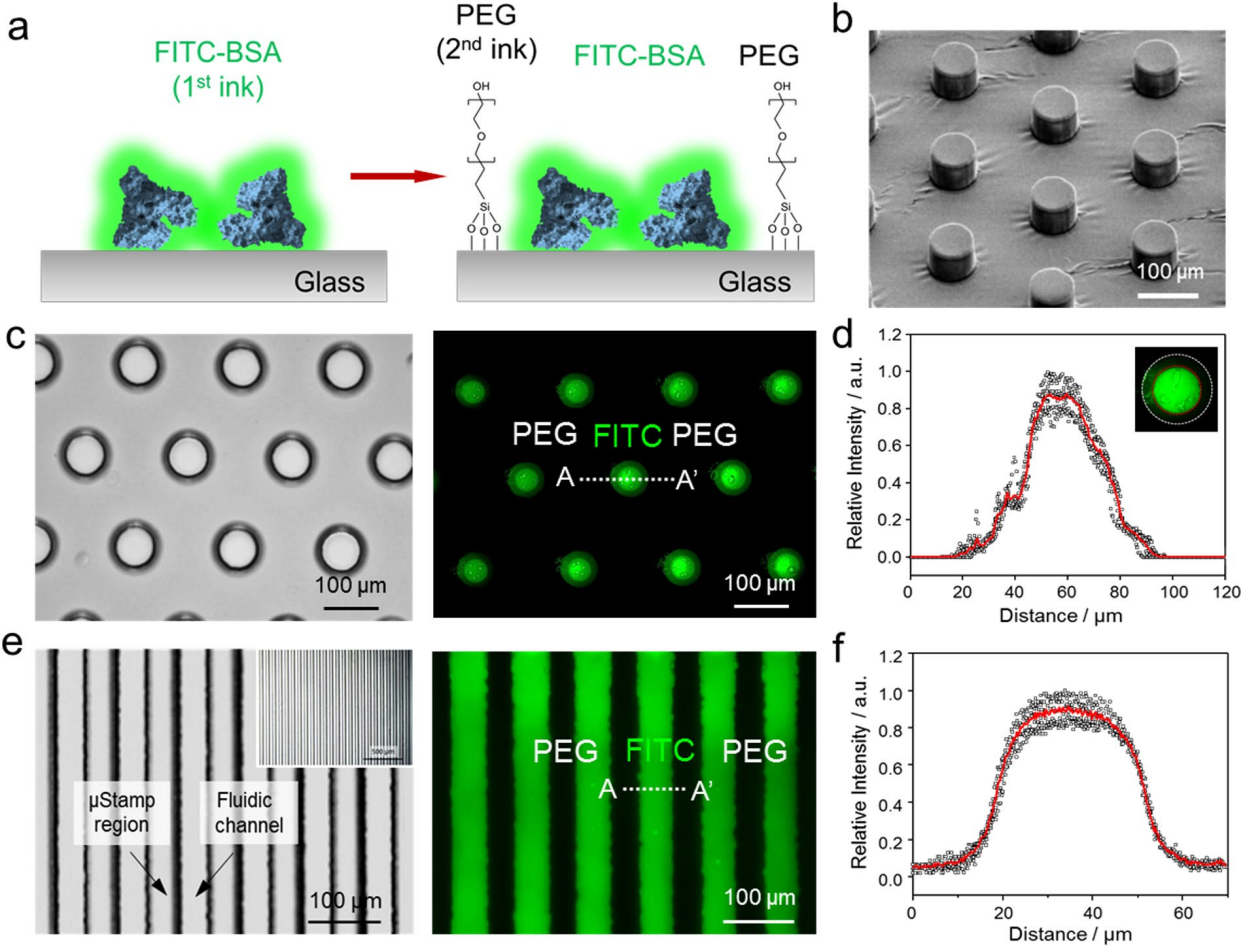
Characterization of PDMS stamp and patterned surface by μCP-DFGP. (**a**) Schematic representation of the sequential μCP-DFGP procedure. (**b**) Scanning electron micrograph of a fabricated PDMS stamp. (**c**) Optical image for a PDMS stamp containing a circular dot array (left) and fluorescent image of patterned FITC-BSA and surrounding PEG-silane (right). (**d**) Line profile showing a clearly defined bi-composite fluorescent pattern. Inset image indicates the lateral speading of patterned 1^st^ ink. (**e**) Optical image for PDMS stamp containing line pattens (left) and fluorescent image for FITC-BSA/PEG-silane line patterns (right). (**f**) Parallel line profile showing a clearly defined bi-composite fluorescent pattern. The lines had a width of 50 μm and a spacing of 30 μm. The scale bars represent 100 μm.

Figure 3 depicts schematics for the patterned BSA-FITC/PEG surface. The surface topography of the PDMS stamp was also examined by a scanning electron microscopy (SEM) as shown in Fig. 3b. The image of SEM shows a regularly spaced and uniformly shaped cylindrical micropilliar. The diameter of bare circular regions in the PDMS stamp was 70 µm (see Supplementary Fig. S1 for the dimension of elastomeric PDMS stamp), revealing that the pattern on the original PDMS stamp has been faithfully transferred to the glass surface. This result indicates that a homogeneous pillar structure was formed and that the pattern has been replicated. Figure 3c shows fluorescent images of patterned circular dots of a FITC-BSA/PEG-silane surface that formed on glass substrate. Using the PDMS stamps, fluorescein isothiocyanate labelled bovine serum albumin (FITC-BSA) (25 µg/ml in PBS), was picked up and transferred onto the glass surface, which offered a convenient way to evaluate the outcome of micropattern through appropriate fluorescence imaging. Then, to introduce the 2^nd^ passivation ink (PEG-silane) inside cavities, we injected the 2^nd^ ink into the inlet of the patterned PDMS stamp, achieving a homogenous distribution by µCP and DFGP. When the high concentration of PEG-silane (100 mM of PEG in ethanol) ink was injected by DFGP, the 2^nd^ PEG-silane ink selectively wetted free cavity regions between the micropillar structures. PEG-silanization of glass surfaces is normally used to increase the attachment stability of biomolecule and prevents the non-specific binding onto a glass and silicon surface^7^. After 1 h incubation, a circular dot array in the desired location was formed which was uniform over the entire patterned area.

As can be seen in Fig. 3d, line intensity profiles across 5 circular dots were used to measure the homogeneity of FITC-BSA patterns. The fluorescence intensity of FITC-BSA was gradually condensed to a core region across each dot by lateral spreading, indicating that free FITC molecules moved inwardly from outer contact areas to the core regions (inset in Fig. 3d). The patterned circular dots were approximately 50 µm in diameter, showing a strong fluorescence intensity (intensity > 0.2), and there was an approximately 20 µm reduction in the diameter of patterned circular dots compare to that of the original PDMS master pillars. This can be attributed to the deformation of PDMS and the incomplete conformal contact between the bottom surface of micropillar and the glass substrate^12^. Thus, it can be assumed that the appearance of FITC-BSA condensation at the core of printed regions is sensitive to the pressure applied during the printing process, which can deform the PDMS stamp. The precision of printing at an edge region of the feature in the PDMS stamp can be improved by producing a stiffer PDMS stamp with sharper edges. Also, although patterning for a single type of molecule is relatively straightforward, the ability to pattern multiple biological/chemical molecules in close proximity without cross-contamination is still challenging. To compare patterned shapes, other PDMS stamp with line patterns was used to print the FITC-BSA on the glass substrate (Fig. 3f). The line array, consisting of parallel lines (dimension for a stamp with parallel lines: 50 µm in width, 30 µm of spacing, 50 µm in height, see Supplementary Fig. S1 for the dimension of elastomeric PDMS stamp) also showed a relatively uniform pattern over the entire patterned area. However, the slightly raised relief features on the unwanted regions were observed due to the deformation of elastomeric PDMS stamp by applying much vertical pressure and lateral spreading of ink. These results demonstrate that clear micropatterned FITC-BSA and PEG-silane SAM were formed on the glass substrate via our sequential µCP-DFGP method.

**Figure 4:**
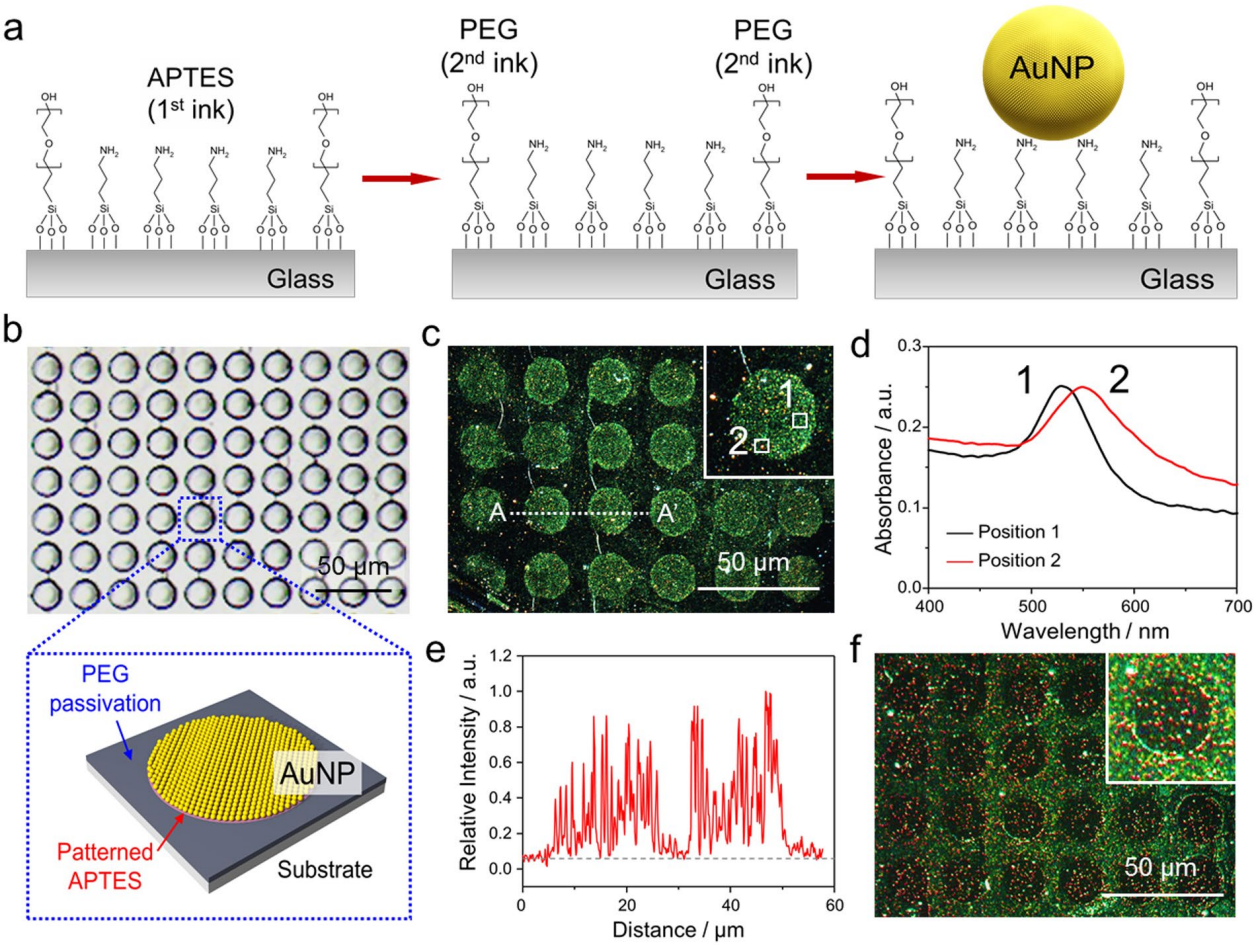
Immobilization of AuNPs on patterned ATPES/PEG-silane bi-composite self-assembled monolayers (SAMs) on a glass slide. (**a**) Schematic illustration of bi-composite patterning via μCP and DFGP. 1^st^ APTES ink was firstly patterned by μCP, then 2^nd^ PEG-silane ink was injected by DFGP. After washing the patterned surface, AuNPs covered the entire patterned surface for 30 s and then were washed. (**b**) optical image of a PDMS stamp for AuNP patterning (top) and schematic diagram of APTES and PEG-silane patterned glass (bottom). (**c**) A dark-field image of AuNP patterning with circular dot patterns. Inserted images represent scattering color with a dark-field microcope. Inset indicates an enlarged dark-field image to display the scattering color. (**d**) Representative scattering spectra. A single bare AuNP (black line, position 1) and aggregated AuNPs due to plasmon coupling via neighbouring AuNPs (red line, position 2) (**e**) Line profiles (**f**) A dark-field image of AuNPs on a reverse bi-composite SAM surface (PEG-silane/APTES). The scale bars represent 50 μm.

Self-assembled colloidal AuNP monolayer patterning by µCP and DFGP was performed as shown in Fig. 4a. The 3-aminopropyltriethoxysilane (APTES, 1^st^ ink) and PEG-silane (2^nd^ ink) were patterned to form a specific linker for AuNP and for passivation of the free surface, respectively. This APTES/PEG-silane was employed to guide the assembly of citrate passivated AuNPs according to their differential affinities for amino (NH^3+^) and hydroxyl groups for APTES and PEG, respectively^6^. Then, the immobilization of colloidal AuNPs (40 nm in diameter) was followed, which benefitted a patterning test due to the homogeneity of colloidal AuNPs in a liquid form^8^. Figure 4b shows an optical image of µstamp of 20 µm in diameter with 5 µm spacing (see Supplementary Fig. S1 for the dimension of elastomeric PDMS stamp). Figure 4c shows dark-field microscopic images of AuNP dot array formed onto the APTES/PEG-saline SAM patterned glass surface. A circular AuNP dot pattern shows that most immobilized AuNPs on the SAM pattern remained intact as a single nanoparticle (position 1). However, as depicted in the inset of Fig. 4c, the plasmonic color in some AuNP regions exhibited a red-shift to orange color (position 2). Figure 4d shows the absorption peak of AuNPs across each position (see inset of Fig. 4c). The maximum spectral intensity of the absorption peak usually depends on the size or the agglomeration of AuNPs. Most AuNPs on the SAM displayed their maximum spectral intensity around 528 nm which indicates that AuNPs were individually immobilized. However, a new plasmonic band at a longer wavelength of 550 nm was observed, which was due to a plasmon coupling phenomenon among neighboring AuNPs, equivalent to an AuNP of 80 nm in diameter. In addition, as shown in Fig. 4e, the line intensity profile for patterned AuNPs on APTES displayed relatively high roughness due to their small size (40 nm in diameter). As a reverse experiment, we switched the sequence as 1^st^ ink of PEG-silane with stamping and 2^nd^ ink with APTES by DFGP (Fig. 4f). Again, correct and desirable patterns were observed, but a few AuNPs in PEG-silane patterned area (PEG stamped circular dot regions) was slightly aggregated with low non-specific AuNP binding onto the PEG surface. It is attributed to the diffusion of APTES ink into the stamped PEG region during the incubation process^35^. To preserve the resolution of pre-patterned features, short incubation time should be enforced when APTES was used as 2^nd^ ink. With this approach via µCP and DFGP, we could achieve a smooth surface with AuNPs and afford good adhesion via APTES pattern to the substrate. This method of nanoparticle assembly offers an attractive option for nanoparticle patterning on a glass/silicon surface, which is relevant in developing biosensors, electronics, and optical devices^6^.

## Conclusions

We described a novel methodology for fabricating a single-layered and multiple patterned surface composed of bi-composite surface patterns by synergistically combining µCP with DFGP. Our facile micropatterning method to simultaneously pattern multiple biochemical molecules in two-dimensional configurations onto substrates. We demonstrated a bi-composite micropatterned surface with different functional molecular inks such as FITC-BSA and PEG-silane for a biomolecule array that directs the biological applications such as the cellular adhesion, guidance for cellular interaction and polarization with other multiple bioactive molecular inks. Also, this technique has the advantage to fabricate the microscale pattern with an assembling of AuNPs for the practical bio-recognition application. As a consequence, our rapid facile patterning methodology by combining µCP with DFGP provides the simplistic way for preparing desired micropatterns without the repeated µCP procedure. In addition, further systematic work will be focused on reducing the size of the printed feature with various functional molecular inks down to the nanoscale for biochip applications.

## Methods

### Materials

FITC-BSA (Sigma) was used without purification. Gold nanoparticles were purchased from BBI (UK). Short-chain poly(ethylene glycol) (PEG) silane ([hydroxy(polyethyleneoxy)propyl] triethoxysilane, Mw: 500–550 Da, 8–12 ethylene oxide repeat units as reported by the manufacturer) was purchased from Gelest (SIH6188.0, Morrisville, PA, USA). Sylgard 184 silicone elastomer kit was purchased from Dow Corning (Midland, MI, USA).

### Fabrication of PDMS stamp

The PDMS stamp for µCP and DFGP were fabricated using the standard soft lithography replica moulding method. Briefly, a mould was created through a single-layer fabrication process using a negative photoresist, SU8-3035 (MicroChem, MA) to form 30 µm thickness on Si wafers. The photoresist was poured onto the wafer at 500 rpm, then the wafer was soft-baked at 65 °C for 5 min and 95 °C for 15 min, followed by UV exposure for 7 s at 9.5 mW/cm^2^ using a mask aligner. The wafer was then baked for 5 min at 65 °C and 10 min at 95 °C, and allowed to cool to room temperature. Finally, the wafer was developed in a SU8 developer for 1 min, rinsed with isopropanol, and blow dried using N_2_. Subsequently, masters were exposed to vapor of (tridecafluoro-1,1,2,2-tetrahydrooctyl)-1-trichlorosilane (Sigma-Aldrich) under vacuum for 12 h to facilitate the release of the PDMS mould after curing.

PDMS (Sylgard 184, Dow Corning) was prepared with a 10:1 mass ratio (base to cross-linker), and degassed in a vacuum chamber for 30 min. Then PDMS prepolymer was poured on the SU8 mould to a thickness of ~ 2 mm, then placed on a dry oven at 80 °C for at least 6 h to ensure complete curing. The master mould was then carefully peeled off from the cured PDMS replica and cut into pieces to use as a stamp. The microfabricated PDMS stamp was characterized by a scanning electron microscope (FE-SEM 7800F Prime, JEOL Ltd, Japan).

### Flow characterization

Before the flow rate characterization, PDMS µStamp degassed in a vacuum chamber at 0.1 MPa for 5 to 15 min that all the air could be removed from the PDMS µStamp. The PDMS devices were then pulled out from the vacuum chamber and places under an iPhone6 because the microscope objective was unable to capture the entire microchannel. The drop of red food coloring dye (20 µl) was then placed directly on the inlet using a micropipette. The iPhone6 was used to acquire time-lapse images of the microfluidic channels until they were completely filled with red food coloring dye, and then ImageJ (NIH) was used for the image anlaysis.

### Microcontact printing

Glass slides were sonicated in isopropanol, ethanol, and deionized (DI) water consecutively for 10 min each, then thoroughly rinsed with DI water, and dried under N_2_ stream. Prior to µCP and DFGP experiment, the µstamp was treated with oxygen plasma for 10 s. This process allowed the stamp surface to become hydrophilic, which ensured homogeneous spreading of the ink. The stamps were prepared fresh prior to use. The oxygen-activated PDMS stamp was immediately inked with FITC-BSA (25 µg/ml in PBS) and then incubated for 10 min to be adsorbed onto the surface of the stamp. Excess ink solution was removed from the PDMS surface with a pipette and the stamp. The stamp was placed onto the glass surface to make a conformal contact between the stamps and the glass substrate. Meanwhile passivation solution such as PEG-silane at 100 mM in ethanol was injected by DFGP. After incubation for 1 h, the micropatterned glass substrate was washed 3 times with PBS and DI water. The patterned surfaces were characterized by a fluorescence microscopy and a dark-field microscope (Olympus IX70, Japan). Images from the fluorescence and dark-field microscope were analyzed with ImageJ software (NIH).

## Electronic supplementary material

SUPPLEMENTARY INFORMATION
