# Supplementary material for: Multifunctional self-assembled monolayers via microcontact printing and degas-driven flow guided patterning
Source: Sci Rep. 2018 Nov 13;8:16763. doi: 10.1038/s41598-018-35195-9 (PMC6233183; doi:10.1038/s41598-018-35195-9)
Supplement: Supplementary file 1 — SUPPLEMENTARY INFORMATION [file 41598_2018_35195_MOESM1_ESM.docx]

SUPPLEMENTARY INFORMATION

Multifunctional self-assembled monolayers via microcontact printing and degas-driven flow guided patterning

Sang Hun Lee^1^, Won-Yeop Rho^2^, Seon Joo Park^3^, Jinyeong Kim^3^, Oh Seok Kwon^3^, Bong-Hyun Jun^2,*^

^1^School of Chemical & Biological Engineering, Seoul National University, Seoul 00826, Republic of Korea

^2^Department of Bioscience and Biotechnology, Konkuk University, Seoul, 05029, Republic of Korea

^3^Harzards Monitoring Bionano Research Center, Korea Research Institute of Bioscience and Biotechnology (KRIBB), Daejeon 34141, Republic of Korea

Author Information:

*Corresponding author. Prof. B. H. Jun, Email: [bjun@konkuk.ac.kr](mailto:bjun@konkuk.ac.kr)


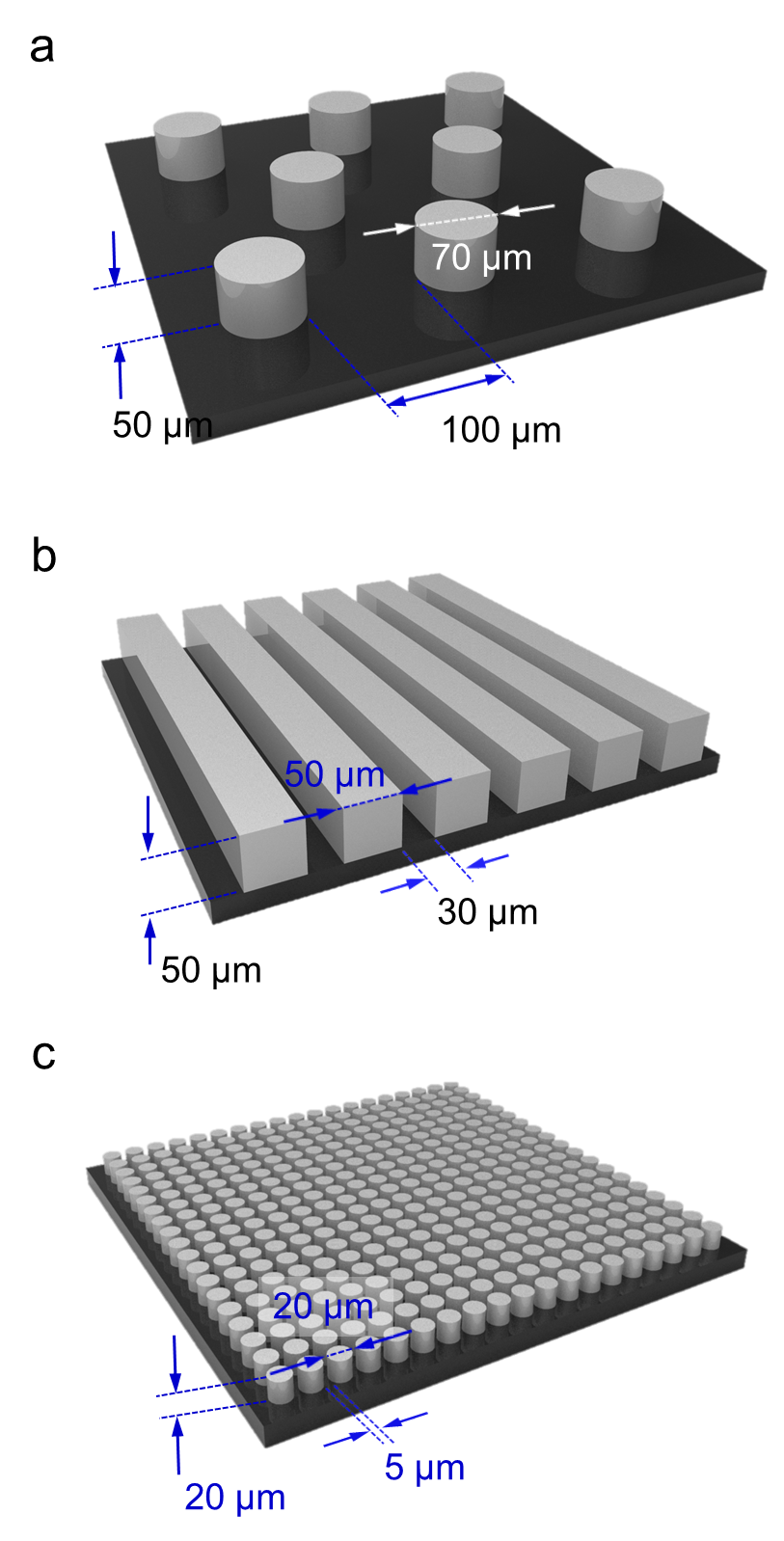


**Supplementary Fig. S1**. Schematic illustrations of the elastomeric PDMS stamps. The different shaped PDMS stamps such as circular dots (a), lines (b), and small dots (c) were used in this work.
